# Biosurfactant-Mediated Inhibition of *Salmonella* Typhimurium Biofilms on Plastics: Influence of Lipopolysaccharide Structure

**DOI:** 10.3390/microorganisms13092130

**Published:** 2025-09-11

**Authors:** Shadi Khonsari, Andrea Cossu, Milan Vu, Dallas Roulston, Massimiliano Marvasi, Diane Purchase

**Affiliations:** 1 Department of Natural Sciences, Faculty of Science and Technology, Middlesex University, London NW4 4BT, UK; s.khonsari@mdx.ac.uk (S.K.); a.cossu@mdx.ac.uk (A.C.); m.vu@mdx.ac.uk (M.V.); 2 Division of Medicine, University College London (UCL), London WC1E 6JF, UK; dallas.roulston@ucl.ac.uk; 3 Department of Biology, University of Florence, Via Madonna del Piano 6, 50019 Sesto Fiorentino, Italy; massimiliano.marvasi@unifi.it

**Keywords:** *Salmonella*, biofilm, lipopolysaccharide, plastic surfaces, surfactin

## Abstract

*Salmonella enterica* subsp. *enterica* serovar Typhimurium is a major foodborne pathogen whose ability to form biofilms contributes to persistent contamination in food-processing and clinical environments. This study investigated the anti-biofilm activity of the biosurfactant surfactin, produced by *Bacillus subtilis*, against *S*. Typhimurium wild type (LT2) and its lipopolysaccharide (LPS)-modified mutants on commonly used plastic surfaces such as polypropylene (PP) and polystyrene (PS). Biofilm formation was quantified using the crystal violet assay, revealing significantly higher biomass on PS compared to PP (*p* < 0.0001). Surfactin at 5 µg/mL was identified as the minimum biofilm inhibitory concentration (MBIC), significantly reducing biofilm formation in the wild-type and LPS mutants *rfaL*, *rfaJ*, *rfaF* (all *p* < 0.0001), and *rfaI* (*p* < 0.01). Further analysis using fluorescence microscopy and SYPRO^®^ Ruby staining confirmed a significant reduction in extracellular polymeric substances (EPSs) on PP surfaces following surfactin treatment, particularly in strains LT2 (*p* < 0.0001), *rfa* (*p* < 0.01), *rfaL* (*p* < 0.0001), *rfaG* (*p* < 0.05), and *rfaE* (*p* < 0.0001). These findings highlight the influence of LPS structure on biofilm development and demonstrate surfactin’s potential as an eco-friendly antimicrobial agent for controlling *S*. Typhimurium biofilms on food-contact surfaces. Analysis of mutants revealed that disruption of the *rfa* gene, which is involved in the biosynthesis of the outermost region of the lipopolysaccharide (LPS), significantly reduced bacterial attachment to polypropylene. This suggests that interactions between the external LPS layer and the plastic surface are important for colonisation. In contrast, mutants in core LPS biosynthesis genes such as *rfaE* and *rfaD* did not show any notable differences in attachment compared to the wild-type strain. This highlights the specific importance of outer LPS components, particularly under surfactant conditions, in mediating interactions with plastic surfaces. This work supports the application of biosurfactants in food safety strategies to reduce the risk of biofilm-associated contamination.

## 1. Introduction

Biofilms are communities of microorganisms, including bacteria, protozoa, archaea, and yeasts, which attach to various surfaces. Around 40–80% of bacterial species on Earth can form biofilms [1]. These communities produce a matrix of polysaccharides, proteins, and extracellular DNA, which holds them together and protects them from environmental stresses [2,3].

Advantages of biofilm structures allow include enhanced survival in nutrient-limited environments, improved nutrient acquisition, increased resistance to biocides, greater productivity, and heightened stability [2,4].

The development of bacterial biofilms involves several stages, including a reversible attachment to surfaces followed by an irreversible attachment through the production of extracellular polymeric substances (EPSs), which results in biofilm maturation and eventual dispersal. Different bacteria exhibit differently articulated regulatory mechanisms during these stages, making understanding biofilm formation a complex and challenging topic [2,5].

The presence of biofilms can have negative impacts, particularly in healthcare settings. Biofilm production on the surfaces of medical devices like catheters and implants can lead to persistent infections and chronic illnesses in affected patients [6,7]. Common biofilm-forming bacteria include *Staphylococcus aureus*, *Staphylococcus epidermidis*, and *Pseudomonas aeruginosa*, which are responsible for many device-related infections [6]. These infections can lead to prolonged hospital stays, additional treatments, the development of antibiotic resistance, and increased healthcare costs.

The US National Institute of Health and the Centres for Disease Control and Prevention report that 80% of microbial diseases are biofilm-related [8]. Among these diseases, *Salmonella enterica* subsp. *enterica* serovar Typhimurium (*S*. Typhimurium), known for its biofilm-forming ability, is particularly problematic in the food industry, leading to microbial contamination and significant financial losses due to food spoilage and potential batch recalls, as well as the need for rigorous cleaning protocols [9]. This dual impact on health and finances underscores the critical need for effective biofilm management.

**Figure 1:**
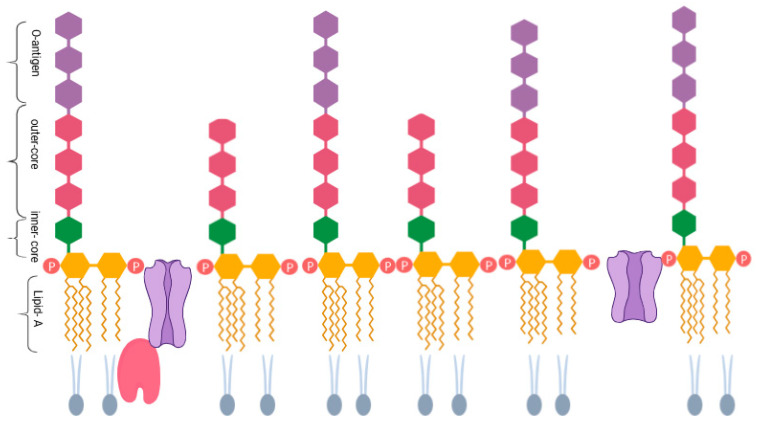
Lipopolysaccharide structure of Gram-negative bacteria, including three regions located on the outer-membrane (OM) LPS. The main component of the OM can change its structure in response to the environment and strongly stimulate the innate immune response, which is also crucial in host–pathogen interactions [12].

The outer membrane of Gram-negative bacteria, including *S*. Typhimurium, contains lipopolysaccharide (LPS). LPS is a large molecule comprising three parts: a lipid A, inner- and outer-core oligosaccharides, and an O-antigen, all connected by covalent bonds [10] (Figure 1). LPS is crucial for maintaining the structural integrity of bacteria and protecting them from chemical stresses. It is the main antigen on the surface of many Gram-negative bacteria, covering up to 80% of the outer membrane in species like *Escherichia coli* and *Salmonella* spp. Additionally, it enhances the external negative charge of the cell membrane, helping to stabilise its structure [11].

While the overall structure of LPS is mainly conserved among the genera of Enterobacteriaceae, there can be variations at the species and subspecies levels [13]. Lipid A is generally conserved within a species, but environmental conditions can influence its regulation. The core oligosaccharide may differ among subspecies, and the O-antigen is the most variable part, with different structures even within subspecies. Some Gram-negative bacteria may lack the O-antigen component [13].

Changes in the length or composition of LPS in Gram-negative bacteria can play a key role in the initial adhesion process and in biofilm formation [14]. In this study, selected *S*. Typhimurium LPS-modified strains were chosen to investigate the role of mutated biosynthesis genes in biofilm formation.

Biofilms pose significant challenges to health because they cause human-related infections which require effective prevention, control, and inhibition strategies. Biosurfactants, which are natural bioactive antimicrobial compounds from fungi, bacteria, and plants, offer possible solutions, with diverse properties suitable for various industrial applications. They exhibit significant biological activities, including antibacterial, antifungal, antiviral, antioxidant, anticancer, and immunomodulatory effects [15]. Over the past two decades, microbial surfactants have been extensively studied for their surface-active properties, enabling their use in agriculture, healthcare, cosmetics, bioremediation, and bioprocessing [16,17]. Biosurfactants are advantageous over synthetic surfactants due to their biodegradability, high specificity, low toxicity, stability under extreme conditions, enhanced efficiency, and low environmental impact, and they can be produced through biotechnological techniques [18,19]. Low-molecular-weight biosurfactants, such as lipopeptides and glycolipids, are particularly promising for pharmaceutical and biotechnological industries [20].

For instance, surfactin, a lipopeptide from *Bacillus subtilis*, is a well-known biosurfactant with broad-spectrum antibacterial properties [21,22]. Its structure includes a ring-shaped cyclic lipopeptide chain and a β-hydroxyl fatty acid chain. Surfactin is notable for its ability to disrupt biofilm formation and effectively remove bacteria from surfaces such as glass, polystyrene, and stainless steel [23]. Based on the study by Mireles II et al., 2001 [24], surfactin was shown to significantly inhibit *S. enterica* biofilm formation on polyvinyl chloride (PVC) and urethral catheter surfaces. The findings demonstrated that surfactin interferes with the early stages of biofilm development, likely by disrupting bacterial adhesion to these medical-grade materials.

This study investigates the impact of LPS and the genes involved in its biosynthesis on biofilm formation of *S*. Typhimurium, using surfactin as a disrupting agent against biofilm formation. To our knowledge, this is the first study to simultaneously examine the impact of specific LPS biosynthesis gene mutations on *S.* Typhimurium biofilm formation and assess the biofilm-disrupting activity of the biosurfactant surfactin, providing an integrative framework for understanding and mitigating biofilm-associated infections. Although there have been studies focusing on surfaces such as PVC associated with medical devices, studies on plastics associated with food preparation have been limited to date. The findings presented here may contribute to developing effective biofilm prevention strategies through biosurfactant-based interventions in the food industry.

## 2. Materials and Methods

### 2.1. S. Typhimurium Strains and Culture Preparation

**Figure 2:**
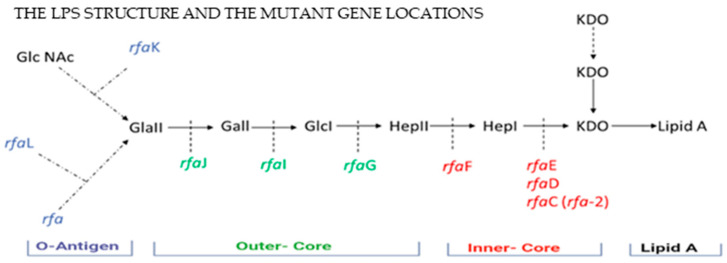
Structure and biosynthesis pathway of lipopolysaccharide (LPS) in *S*. Typhimurium, illustrating the major LPS components (in black) and the specific biosynthesis genes (in different colours) involved at each stage of LPS assembly (Modified from [25]). The biosynthesis involves sequential addition of sugars such as GlcNAc (N-acetylglucosamine), Gal (galactose), Glc (glucose), Hep (heptose), and KDO (3-deoxy-D-manno-oct-2-ulosonic acid) to a Lipid A anchor. The LPS-modified mutants used in this study are indicated in different colours based on their LPS locations: *rfa*, *rfaL*, *rfaK*, *rfaJ*, *rfaI*, *rfaG*, *rfaF*, *rfaE*, *rfaD*.

Selected strains of *S.* Typhimurium were obtained from the *Salmonella* Genetic Stock Centre (SGSC, Canada). These strains include *S*. Typhimurium WT (LT2) and LPS-modified mutants *rfa*, *rfaL*, *rfaK*, *rfaJ*, *rfaI*, *rfaG*, *rfaF*, *rfaE*, and *rfaD,* colour-coded by region (Figure 2). Mutants used in this study are colour-coded by region, allowing investigation of how specific LPS structural changes affect bacterial behaviour in the formation of biofilms.

### 2.2. Biosurfactant Inhibition Efficiency Test

The minimum biofilm inhibitory concentration (MBIC) is defined as the lowest concentration of the biosurfactant required to produce a statistically significant reduction in biofilm formation compared to the untreated control [26]. To determine the MBIC of surfactin for inhibiting biofilm formation, a concentration gradient ranging from 100 µg/mL to 0.05 µg/mL (100, 70, 50, 5.0, 2.5, 0.5, and 0.05 µg/mL) was prepared using commercially available surfactin derived from *Bacillus subtilis* (Sigma-Aldrich; Dorset, UK). The compound was dissolved in absolute ethanol (Fisher Scientific; Leicestershire, UK) and further diluted (dH2O) to desirable concentrations using the dissolved stock solution. The working surfactin concentration solutions (all reconstituted in 10% ethanol) were applied to overnight cultures (standardised according to the 0.5 McFarland standard at OD_600_) of *S.* Typhimurium WT (LT2) and selected LPS-modified mutants grown in 5 mL of lysogeny broth (LB) at 37 °C (Fisher Scientific; Leicestershire, UK). For the biofilm activity assay, 20 µL of each overnight culture was inoculated into individual wells of a sterile 96-well flat-bottom polystyrene microtiter plate (Fisher Scientific; Leicestershire, UK). Each well was then filled with 200 µL of colonisation factor antigen (CFA) medium, composed of 10 g/L casamino acids, 1.5 g/L yeast extract, 0.4 mM MgSO_4_, and 0.4 mM MgCl_2_ (Oxoid; Hampshire, UK). Plates were incubated at 37 °C for 72 h under static conditions to allow for biofilm development. Following incubation, planktonic cells were removed by washing the wells three times with distilled water (dH_2_O). Biofilms were stained by adding 30 µL of 1% crystal violet solution (Acros Organics; Fairlawn, NJ, USA) to each well and incubating for 20 min at room temperature. Excess stain was removed, and the wells were washed twice with dH_2_O. To solubilise the bound dye, 200 µL of 30% acetic acid (Fisher Scientific; Leicestershire, UK) was added to each well [27]. The experiment was conducted in 5 technical replicates (wells in a microplate) and 3 biological replicates (microplates).

Biofilm biomass was quantified by measuring absorbance at 595 nm using a FLUOstar Omega microplate reader (BMG Labtech, v5.11; De Meern, The Netherlands).

### 2.3. Bactericidal and Bacteriostatic Activity Test

To evaluate the bacteriostatic effect of surfactin, *S.* Typhimurium WT (LT2) was cultured overnight in 5 mL of LB at 37 °C. The culture was then serially diluted in sterile saline solution (0.154 M NaCl) and standardised to a 0.5 McFarland turbidity standard, corresponding to an optical density at 600 nm (OD_600_) of between 0.08 and 0.1.

Commercially available 4.5% bleach (Domestos; Surrey, UK), containing sodium hypochlorite (NaOCl) as the active ingredient along with non-ionic surfactants, soap, perfume, amines, coco alkyldimethyl N-oxides, sodium hydroxide, and 1-hexadecanaminium compounds, was used as the positive bactericidal control. For specificity, sodium hypochlorite was considered the active antimicrobial component.

Surfactin was added to the standardised bacterial suspension at a final concentration of 5 µg/mL and mixed thoroughly. A 10 µL aliquot of the mixture was immediately plated on xylose lysine deoxycholate (XLD) agar (Oxoid; Hampshire, UK) and incubated at 37 °C for 24 h to establish the baseline colony count (CFU/mL at T_0_).

The remaining diluted cultures were maintained at 37 °C for 4 h under rapid shaking conditions (100 rpm in orbital incubator) to prevent cell clumping. After incubation (T_1_; 4 h after the application of surfactin), 10 µL samples were again plated on XLD agar and incubated at 37 °C for 24 h. Colony counts from T_1_ were compared to those from T_0_ and the untreated control to determine the inhibitory (bacteriostatic) or lethal (bactericidal) effects of surfactin. The experiment was conducted in 3 biological replicates (agar plates).

### 2.4. Quantification of S. Typhimurium WT LT2 and LPS-Modified Mutants’ Biofilm Formation on Plastic Surfaces in the Presence or Absence of Surfactin

To investigate the influence of different plastic surfaces on biofilm formation, two types of 96-well microtiter plates, polypropylene (PP) and polystyrene (PS), were used (Thermo Scientific; Newport, UK). The biosurfactant surfactin was prepared at a concentration of 5 µg/mL by dissolving it in absolute ethanol (Fisher Scientific; Leicestershire, UK). This solution was added to the wells of each plate and allowed to evaporate completely at room temperature, ensuring uniform coating of the well surfaces.

Following evaporation, 20 µL of overnight bacterial cultures of selected LPS-modified *S*. Typhimurium mutants were added to each well, along with 200 µL of colonisation factor antigen (CFA) liquid medium. The plates were incubated at 37 °C for 72 h under static conditions to allow for biofilm development.

After incubation, biofilm formation on both PP and PS surfaces in the presence of surfactin was assessed using the crystal violet (CV) staining method, as described above; wells without surfactin treatment served as non-treated controls for comparative analysis. The experiment was conducted in 5 technical replicates (wells in a microplate) and 3 biological replicates (microplates).

### 2.5. Quantification of EPS Using Fluorescence Microscopy

#### 2.5.1. Culture Sample Preparations

*S.* Typhimurium WT (LT2) and LPS-modified mutants *rfaL*, *rfa*G, and *rfa*E, each representing different modifications in the lipopolysaccharide (LPS) chain lengths, were selected for this study. The strains were cultured in nutrient broth (NB) (Oxoid; Hampshire, UK), and incubated at 37 °C for 24 h. Following incubation, the bacterial suspensions were adjusted to a final microbial concentration of 1.0 × 10^7^ CFU/mL using sterile NB.

Sterile polypropylene (PP) coupons (1 cm × 1 cm; Essential Arts Products Ltd., Wilmington, UK) were placed individually into wells of sterile 12-well polystyrene plates. Each well was filled with 2.5 mL of the prepared bacterial suspension. To assess the antimicrobial effects of the biosurfactant, 45 µL of surfactin solution at a final concentration of 5 µg/mL was used. The same volumes of commercially available sodium hypochlorite solution (final concentration: 4.5% *v*/*v*) and dH_2_O served as bactericidal control and untreated control, respectively.

Biofilm formation was carried out by incubating the plates containing the surface coupons and treatment suspensions at 37 °C for 72 h under static conditions. To maintain nutrient availability and ensure consistent treatment efficacy, the medium in each well was carefully replaced with fresh nutrient broth (NB) containing the respective treatments after 24 h of incubation.

#### 2.5.2. Fluorescence Microscopy

Following a 72-h incubation period, polypropylene (PP) coupons were retrieved and gently rinsed with dH_2_O to remove non-adherent cells. The coupons were then stained with 200 µL of FilmTracer™ SYPRO™ Ruby Biofilm Matrix Stain and incubated in the dark at room temperature for 30 min. After a final rinse with dH_2_O, the coupons were mounted on microscope slides and covered with coverslips for imaging.

Fluorescence microscopy was performed using an Olympus BX51 microscope equipped with a red dichroic filter (excitation: 460–490 nm; emission: 590 nm). Initial visualisation was conducted using LightField lenses (Exposure: 132.368; Gain: 140), followed by imaging with the red filter (Exposure: 2000; Gain: 129). Images were captured using an AmScope MU900-CK 9MP digital camera. To qualitatively assess EPS biofilm formation, five regions per coupon (four edges and the centre) were imaged for each strain under both treated and untreated conditions. EPS intensity was quantified using ImageJ-Fiji software (v1.54f), an open-source platform based upon ImageJ [28]. The experiment was conducted in triplicate (biological, 3 coupons), and signal intensity was taken from 5 areas per coupon (total of 15 replicates).

### 2.6. Statistical Analysis

All statistical analyses were performed using GraphPad Prism version 7.04 (GraphPad Software, San Diego, CA, USA). Data from the inhibition efficiency assay, bactericidal and bacteriostatic activity tests, and biofilm quantification on plastic surfaces were analysed for statistical significance. Comparisons between treated and untreated control groups were conducted using Student’s *t*-tests with a 95% confidence interval. For experiments involving multiple treatment concentrations, a one-way analysis of variance (ANOVA) followed by Tukey’s post hoc test was applied to compare the treated *S*. Typhimurium WT (LT2) groups with the untreated control. A *p*-value ≤ 0.05 was considered statistically significant.

Image analysis was conducted using ImageJ-Fiji (v1.54f), and fluorescence intensity data (n = 13–15) were statistically analysed using GraphPad Prism. A one-way ANOVA with Tukey’s post hoc test was applied to determine significance, and outliers were identified and excluded using GraphPad’s built-in outlier calculator.

Chatgpt was used to improve the English language for writing the article.

## 3. Results

### 3.1. Comparison of Biofilm-Forming Ability of S. Typhimurium WT (LT2) and Mutants on Plastic Surfaces

**Figure 3:**
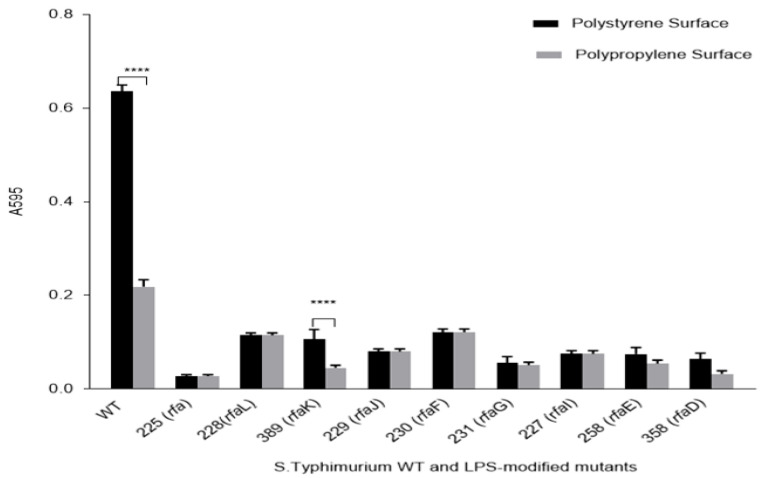
Biofilm formation by *S*. Typhimurium WT (LT2) and ten LPS-modified gene mutants with deletion of genes located on different parts of LPS. Mutants are presented in order of their location on LPS: O-antigen genes *rfa*, *rfaL*, *rfaK*; inner-core genes *rfaJ*, *rfaG*, *rfaI*,; outer-core genes *rfaF*, *rfaE*, *rfaD*. ****: *p* < 0.0001.

The biofilm-forming ability of *S.* Typhimurium WT LT2 and selected LPS-modified mutants was evaluated on the two plastic surfaces of PP and PS, using the CV assay. Quantitative analysis revealed that both the WT strain and the mutant *rfaK* exhibited significantly higher biofilm formation on PS compared to PP surfaces (*p* < 0.0001) (Figure 3). These findings indicate a surface-dependent variation in biofilm development, with PS promoting greater bacterial adhesion and accumulation than PP.

### 3.2. Biosurfactant Inhibition Test and Bactericidal Properties

**Figure 4:**
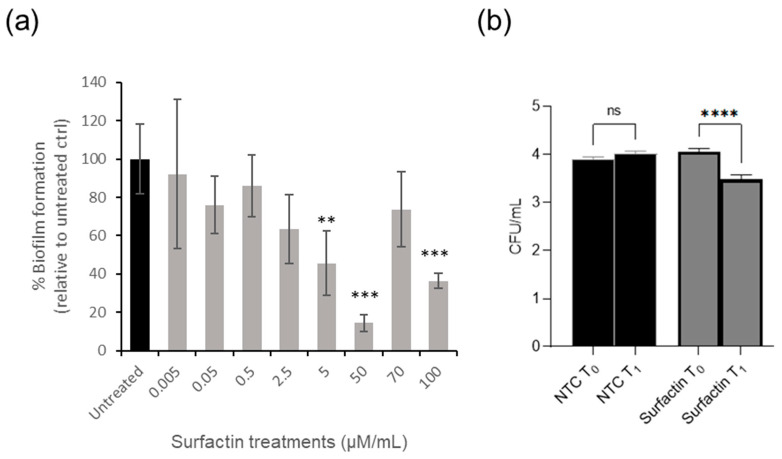
(**a**) Inhibition of *S*. Typhimurium LT2 biofilm formations on PS surface by surfactin at a range of concentrations, from the lowest concentration of 0.005 µg/mL to the highest concentration of 100 µg/mL, data presented as % biofilm formation relative to untreated control. (**b**) *S*. Typhimurium LT2 was grown on XLD plates in the presence of 5 µg/mL surfactin. NTC indicates the LT2 has no added biosurfactant (negative control); T_0_ is the initial time when biosurfactant has been applied to the bacterial cells grown on XLD, and T_1_ is 4 h after the application of biosurfactant. **: *p* < 0.01, *** *p* < 0.001 ****: *p* < 0.0001, ns: no significant difference.

The inhibition efficiency of various concentrations of surfactin (ranging from 0.005 µg/mL to 100 µg/mL) was examined. The CV assay demonstrated that biofilm formation by *S.* Typhimurium (LT2) was inhibited by surfactin at 5 µg/mL as the MBIC, with a significant (*p* < 0.0001) biofilm inhibition. Additionally, tested concentrations of surfactin above 50 µg/mL did not significantly affect biofilm formation when compared to the untreated control (Figure 4a).

Surfactin at 5 µg/mL was selected for further evaluation of its bactericidal activity using colony counts on XLD plates. After 4 h (T_1_) of surfactin treatment at 5 µg/mL, there was a significant reduction (*p* < 0.0001) of colony-forming units compared to the initial time point (T_0_) (Figure 4b).

### 3.3. Quantification of Biofilm Formations of Selected Strains in the Presence of Surfactin on Plastic Surfaces

**Figure 5:**
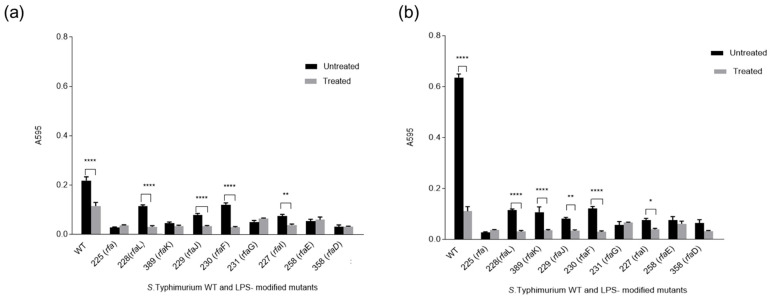
Biofilm formation inhibition in *S*. Typhimurium WT and mutants on polypropylene (PP) (**a**) and polystyrene (PS) (**b**) surfaces in the presence of 5 µg/mL. *: *p* < 0.05, **: *p* < 0.01, ****: *p* < 0.0001.

A selected concentration of surfactin (5 µg/mL) was used to treat the *S*. Typhimurium WT LT2 and the mutants grown on PS (Figure 5a) and PP (Figure 5b) surfaces. Notable suppression of biofilm formation by the treatment was observed in *S*. Typhimurium WT LT2 (*p* < 0.0001) and the following LPS-modified mutants: *rfal*, *rfaJ*, *rfaF* (*p* < 0.0001), and *rfaI* (*p* < 0.01), on PP surfaces. A similar trend was observed on the PS surface, where the treatment inhibited biofilm formation among the same strains: LT2 (WT), *rfaL*, *rfaF* (*p* < 0.0001), *rfaJ* (*p* < 0.01), *rfaI* (*p* < 0.05), and, additionally, in *rfaK* (*p* < 0.0001).

### 3.4. Measuring the EPS of Biofilms Using Fluorescence Microscopy

A previous experiment (Figure 4) demonstrated that *S.* Typhimurium WT LT2 and LPS-modified mutants formed less biofilm on PP surfaces compared to PS surfaces, regardless of biosurfactant treatment. Given PP’s relevance in food processing environments, it was selected for further investigation into biofilm matrix composition and disruption.

**Figure 6:**
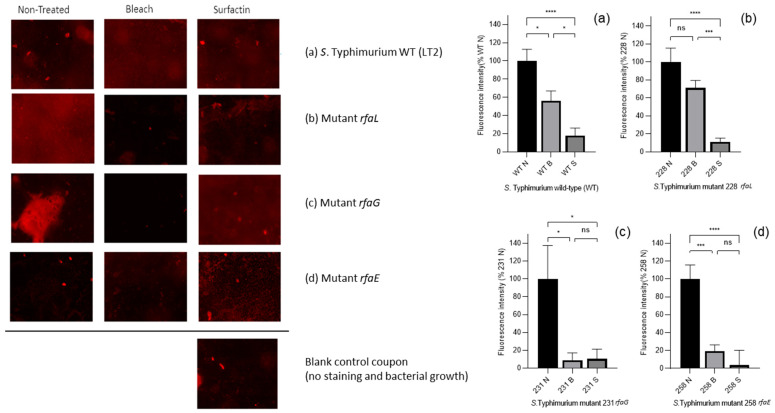
The EPS biofilms of strains formed on PP coupon surfaces, untreated and treated with the antimicrobial agents bleach and surfactin (from left to right), stained with FilmTracer^TM^ SYPRO^TM^ Ruby Biofilm Matrix Stain observed under Olympus Bx51 Fluorescence Microscope; images obtained using AmScope MU900-CK Microscope Digital Camera (20×). Blank stained coupon used as the control to observe the coupon surface without biofilm cell. Representative images of n = 15. Fluorescence intensity (%) of EPS measured with Fiji. One-way ANOVA reveals significant reduction of EPS treated with bleach and surfactin; * *p* < 0.05, *** *p* < 0.001, **** *p* < 0.0001, ns: no significant difference. Graphs/Images: (**a**) *S*. Typhimurium WT, (**b**) mutant *rfaL*, (**c**) mutant *rfaG*, (**d**) mutant *rfaE*. Each bar from left to right: non-treated (N), bleach-treated (B), and surfactin-treated (S).

Statistical analysis using one-way ANOVA revealed significant reductions in EPS across all treated strains compared to untreated controls. The most pronounced reductions (*p* < 0.0001) were observed in the wild-type LT2 (Figure 6a), as well as in mutants *rfaL* (Figure 6c) and *rfaE* (Figure 6d) following surfactin treatment. These findings confirm the efficacy of surfactin in disrupting the biofilm matrix on PP surfaces.

## 4. Discussion

This study examined the biofilm-forming capabilities of *S.* Typhimurium WT (LT2) and LPS-modified mutants on two commonly used plastic surfaces, PS and PP, and evaluated the inhibitory effects of the biosurfactant surfactin. The findings revealed that the wild-type strain formed significantly more biofilm than the LPS mutants, with greater accumulation on PS than PP (Figure 3). These results are consistent with a previous study [29], which reported reduced biofilm formation by *E. coli* on PP compared to PS.

The observed differences in biofilm formation between PS and PP surfaces can be attributed to variations in their physicochemical properties, particularly surface roughness and hydrophobicity. PS is generally more hydrophobic and exhibits a slightly higher surface roughness compared to PP, with average roughness (Ra) values of 0.113 µm and 0.101 µm, respectively [30]. These roughness values, rather than indicating material thickness, reflect the microscale texture of the surfaces, which can influence bacterial adhesion and subsequent biofilm development [31]. The presence of phenyl groups in PS further promotes bacterial attachment, while its negative surface charge may facilitate electrostatic interactions with bacterial cell walls [32,33]. In contrast, PP’s smoother and less hydrophobic surface offers fewer anchoring points, resulting in weaker bacterial adhesion and reduced biofilm development.

These surface–bacteria interactions are critical, especially in the context of food safety, as biofilms on plastic surfaces can increase microbial persistence and resistance to cleaning agents. This is particularly concerning for Gram-negative bacteria like *S*. Typhimurium, which are known for their robust biofilm-forming abilities and resistance to environmental stressors [34,35,36].

To address this challenge, the study focused on optimising the application of surfactin, a potent biosurfactant, for the inhibition of biofilm formation. A concentration of 5 µg/mL was identified as the MBIC, demonstrating the most consistent and effective suppression of biofilm development on both PS and PP surfaces (Figure 4a). This aligns with findings of previous studies which also indicated complete biofilm inhibition at this concentration [24]. Notably, surfactin demonstrated bactericidal activity against *S*. Typhimurium under the tested conditions, further supporting its antimicrobial potential (Figure 4b).

Interestingly, higher concentrations of surfactin (70–100 µg/mL) resulted in reduced efficacy. This may be due to micelle formation [37], which decreases the compound’s bioavailability (Figure 5a). At these concentrations, surfactin molecules tend to self-assemble into micelles, sequestering the active monomers and thereby limiting their interaction with bacterial cell surfaces and biofilm matrices [37]. Other contributing factors may include stress-induced biofilm gene expression and surface conditioning effects, which can alter bacterial adhesion dynamics and reduce treatment effectiveness [38].

To further characterise surfactin’s impact, the study evaluated EPS, a key structural component of biofilms, on PP surfaces. Fluorescence microscopy using SYPRO Ruby staining revealed that surfactin significantly reduced EPS in the WT strain and most LPS mutants, particularly in mutants *rfaE* (Figure 6d) and *rfaG* (Figure 6c). These reductions were comparable to those observed with bleach, a conventional oxidising agent, especially in strains with shorter LPS chains, which likely have thinner EPS layers and are more susceptible to penetration and disruption [39].

This study demonstrates that surfactin significantly reduces EPS in both wild-type and LPS mutant strains of *S.* Typhimurium, with the most pronounced effects being observed in mutants *rfaE* and *rfaG*. These findings highlight the critical role of LPS core structure in maintaining biofilm integrity and resistance to disruption.

The *rfaE* gene is essential for the biosynthesis of heptose sugars in the inner core of LPS, and *rfaG* encodes a glucosyltransferase responsible for adding glucose residues to the outer core. Mutations in these genes result in truncated LPS structures, which compromise the integrity of the outer membrane and alter surface characteristics such as charge and hydrophobicity [40,41]. These structural deficiencies likely increase the permeability of the bacterial envelope, making the biofilm matrix more susceptible to surfactin’s amphiphilic and membrane-disrupting properties [42,43].

Previous studies have shown that *rfaG* mutants exhibit significantly reduced biofilm formation and motility, supporting the idea that LPS core sugars are essential for surface attachment and biofilm maturation [38]. Similarly, defects in LPS biosynthesis have been associated with increased susceptibility to antimicrobial agents [44,45,46,47]. The enhanced sensitivity of *rfaE* and *rfaG* mutants to surfactin observed in this study suggests a synergistic effect, where LPS truncation facilitates biosurfactant penetration and EPS destabilisation, leading to biofilm detachment and structural collapse [47,48].

Surfactin is known for its potent surface-active and antimicrobial properties. Its mechanism of action involves disruption of hydrophobic interactions, insertion into lipid bilayers, and alteration of membrane permeability, which collectively compromise the integrity of the biofilm matrix [41,47,49]. Its ability to disrupt biofilms has been attributed to its interaction with membrane lipids and interference with cell-to-cell adhesion [47]. The pronounced effect of surfactin on LPS-deficient mutants suggests that intact LPS structures may act as a protective barrier, shielding the biofilm matrix from biosurfactant action. This finding supports that LPS mutants, particularly those with deep-rough phenotypes, exhibit increased membrane permeability and reduced resistance to environmental stressors [50].

While the precise molecular interactions between surfactin and LPS structures remain to be fully elucidated, the observed EPS reductions affirm surfactin’s efficacy in disrupting biofilm architecture. Although confocal laser scanning microscopy (CLSM) is the preferred method for biofilm imaging, fluorescence microscopy was employed due to safety constraints [51]. Despite its limitations, this method provided sufficient resolution and sensitivity for quantitative EPS analysis, with careful calibration to minimise photobleaching and phototoxicity [52].

The gene *rfa* is involved in the most external part of the LPS structure and appears to play a key role in attachment to polypropylene (PP). This might be due to interactions between the plastic surface and the outer LPS layer [2]. Interestingly, this finding was somewhat unexpected: it was initially hypothesised that mutations affecting the inner core would have a more pronounced effect on the entire LPS chain integrity and, consequently, on the overall surface attachment. A similar trend was observed for other genes involved in the outer and inner core, including those encoding components of the O-antigen. In contrast, mutations in *rfaE* and *rfaD*, which are located at the base of the LPS biosynthesis pathway, showed no observable phenotype on either the PP or PS surfaces, even in the presence of 5 µg/mL surfactant. These results suggest that the outermost components of the LPS chain are most critical for plastic interaction in the presence of surfactant. This interpretation is supported by the fluorescence microscopy data, which showed reduced biofilm formation in *rfaG* and *rfaL* mutants.

Surfactin, at its MBIC of 5 µg/mL, effectively inhibited biofilm formation on plastic surfaces, and demonstrated multifunctional potential. This finding is supported by pendant drop tensiometry experiments [53,54] which showed a significant reduction in interfacial tension, with surfactin lowering surface tension to approximately 27 mN/m. Additionally, pH stability assays [44,55] confirmed that surfactin retained its surface-active properties and biofilm inhibition capacity across a broad pH range (pH 4–9), highlighting its robustness under variable environmental conditions. These properties make surfactin a promising candidate for food safety applications, including microbial control and food emulsion stabilisation.

## 5. Conclusions

This study provides novel insights into the biofilm-forming behaviour of *S.* Typhimurium wild type (LT2) and LPS-modified mutants on plastic surfaces commonly used in the food industry, with a particular focus on the anti-biofilm potential of the biosurfactant surfactin. It was demonstrated for the first time that the wild-type strain forms significantly more robust biofilms than LPS mutants on both PS and PP surfaces, highlighting the critical role of intact LPS structures in biofilm development, adhesion, and stability.

Among the tested surfaces, PP supported significantly less biofilm formation than PS. These findings suggest that PP is a more suitable material for food-contact applications where biofilm prevention is essential.

Surfactin, at a low concentration of 5 µg/mL, was shown to be highly effective in reducing biofilm formation and EPS production in both wild-type and selected LPS mutant strains. This confirms surfactin’s potential as an eco-friendly, biodegradable antimicrobial agent for use on plastic surfaces, particularly PP, to mitigate biofilm-associated contamination risks.

The study revealed that mutations in specific LPS biosynthesis genes significantly impacted biofilm formation. These genetic alterations were associated with reduced biofilm biomass and altered surface-associated behaviour, suggesting that components of the LPS core may play a role in biofilm stability. These findings highlight potential targets for future biofilm control strategies. By investigating the early-stage inhibitory effects, crucial for strategies aimed at preventing biofilm establishment, this research highlights the importance of LPS integrity, surface material properties, and biosurfactant application in shaping biofilm formation by *S*. Typhimurium. Our research supports the development of surfactin-based interventions for biofilm control in food processing environments. Future studies should explore mechanisms of biofilm disruption as a second step in biofilm control and utilise a multi-omics (genomic, transcriptomic, proteomic) approach to elucidate further surfactin’s mechanism of action and its broader impact on biofilm architecture and bacterial physiology.

## Data Availability

The original contributions presented in this study are included in the article. Further inquiries can be directed to the corresponding author.
